# Qualitative and Quantitative Analysis of the Major Constituents in Chinese Medical Preparation Lianhua-Qingwen Capsule by UPLC-DAD-QTOF-MS

**DOI:** 10.1155/2015/731765

**Published:** 2015-01-08

**Authors:** Weina Jia, Chunhua Wang, Yuefei Wang, Guixiang Pan, Miaomiao Jiang, Zheng Li, Yan Zhu

**Affiliations:** ^1^Tianjin Key Laboratory of Modern Chinese Medicine, Tianjin University of Traditional Chinese Medicine, Tianjin 300193, China; ^2^Tianjin Key Laboratory of TCM Chemistry and Analysis, Tianjin University of Traditional Chinese Medicine, Tianjin 300193, China; ^3^Research and Development Center of Traditional Chinese Medicine, Tianjin International Joint Academy of Biotechnology & Medicine, Tianjin 300457, China

## Abstract

Lianhua-Qingwen capsule (LQC) is a commonly used Chinese medical preparation to treat viral influenza and especially played a very important role in the fight against severe acute respiratory syndrome (SARS) in 2002-2003 in China. In this paper, a rapid ultraperformance liquid chromatography coupled with diode-array detector and quadrupole time-of-flight mass spectrometry (UPLC-DAD-QTOF-MS) method was established for qualitative and quantitative analysis of the major constituents of LQC. A total of 61 compounds including flavonoids, phenylpropanoids, anthraquinones, triterpenoids, iridoids, and other types of compounds were unambiguously or tentatively identified by comparing the retention times and accurate mass measurement with reference compounds or literature data. Among them, twelve representative compounds were further quantified as chemical markers in quantitative analysis, including salidroside, chlorogenic acid, forsythoside E, cryptochlorogenic acid, amygdalin, sweroside, hyperin, rutin, forsythoside A, phillyrin, rhein, and glycyrrhizic acid. The UPLC-DAD method was evaluated with linearity, limit of detection (LOD), limit of quantification (LOQ), precision, stability, repeatability, and recovery tests. The results showed that the developed quantitative method was linear, sensitive, and precise for the quality control of LQC.

## 1. Introduction

Lianhua-Qingwen capsule (LQC), developed from the two classical traditional Chinese medicine (TCM) formulae* Maxing-Shigan-Tang* and* Yinqiao-San* which have a long history of clinical application in the treatment of influenza [1], is a commonly used Chinese medical preparation to treat viral influenza and especially played an important role in the fight against severe acute respiratory syndrome (SARS) in 2002-2003 in China [2]. LQC is composed of 11 herbs including Fructus Forsythiae (Lianqiao), Flos Lonicerae Japonicae (Jinyinhua), Herba Ephedrae (Mahuang), Semen Armeniacae Amarum (Kuxingren), Radix Isatidis (Banlangen), Rhizoma Dryopteridis Crassirhizomatis (Mianmaguanzhong), Herba Houttuyniae (Yuxingcao), Herba Pogostemonis (Guanghuoxiang), Radix* et* Rhizoma Rhei (Dahuang), Radix* et* Rhizoma Rhodiolae Crenulatae (Hongjingtian), and Radix* et* Rhizoma Glycyrrhizae (Gancao), along with menthol and a traditional Chinese mineral medicine, Gypsum Fibrosum (Shigao). According to previous reports, LQC has a good clinical effect on influenza with the symptoms of high fever, aversion to cold, headache, pharyngalgia, cough, sneezing, muscle ache, and so on [3]. Modern pharmacological studies have shown that LQC also has the antiviral, antibacterial, and anti-inflammatory activities [4, 5]. Recently, the study on its bioactive ingredients and molecular mechanism of action has been gradually reported as well [6].

Although some preliminary analytical methods have been developed for the quality control for LQC, including thin layer chromatography (TLC) [7], high performance liquid chromatography (HPLC) [8, 9], micellar electrokinetic capillary chromatography (MEKC) [10], and liquid chromatography tandem mass spectrometry (LC-MS/MS) [11], no systematical and comprehensive study on the chemical profiling and quality control method for LQC has been reported so far. For a classical and complex Chinese medical preparation, the comprehensive quality evaluation method should be based on its multiple chemical constituents. Therefore, it is necessary to develop a rapid and sensitive method to identify and quantify the chemical constituents in LQC, which will be beneficial to investigate the effectiveness and evaluate the quality of LQC.

In this study, a reliable, sensitive, and simple ultraperformance liquid chromatography coupled with diode-array detector and quadrupole time-of-flight mass spectrometry (UPLC-DAD-QTOF-MS) method which was more systematical and comprehensive than the earlier ones was established for characterization and quantification of the major chemical constituents of LQC. A total of 61 compounds were unambiguously or tentatively identified by comparing the retention times, exact molecular masses, and MS/MS spectral data with reference compounds or literature data. Furthermore, twenty-seven compounds were confirmed by comparing with the standards. Among them, twelve representative compounds were quantified as chemical markers in quantitative analysis, including salidroside, chlorogenic acid, forsythoside E, cryptochlorogenic acid, amygdalin, sweroside, hyperin, rutin, forsythoside A, phillyrin, rhein, and glycyrrhizic acid. This is the first systematical and comprehensive study on the qualitative and quantitative analysis of LQC.

## 2. Experimental

### 2.1. Reagents, Chemicals, and Materials

Methanol and acetonitrile (HPLC grade) were purchased from Sigma Aldrich (St. Louis, MO, USA). Formic acid (HPLC grade) was purchased from Tianjin Damao chemical reagent factory (Tianjin, China). Water (HPLC grade) for UPLC analysis was produced by the Milli-Q water purification system (Millipore, USA). Salidroside, chlorogenic acid, forsythoside E, cryptochlorogenic acid, amygdalin, sweroside, hyperin, rutin, forsythoside A, phillyrin, rhein, and glycyrrhizic acid were purchased from Sigma Aldrich (St. Louis, MO, USA). The purity of standard substances was above 98%. Ten batches of LQC were provided by Shijiazhuang Yiling Pharmaceutical Co., Ltd. (Shijiazhuang, China).

### 2.2. UPLC Analysis

The UPLC analysis was performed on a Waters ACQUITY UPLC instrument (Waters Corporation, MA, USA) coupled with a binary pump, a sample manager, an autosampler, a column compartment, and diode-array detector (DAD). The separation of samples was performed on a Waters ACQUITY UPLC BEH C_18_ (100 × 2.1 mm, 1.7 *μ*m) column with the column temperature at 50°C. The analysis was completed in 30 min with a gradient elution of 0.1% formic acid aqueous solution (A) and methanol (B) at the flow rate of 0.3 mL/min. The gradient program was designed as follows: 0–11 min, 5–35% B; 11–18 min, 35–55% B; 18–22 min, 55–75% B; 22–24 min, 75–90% B; 24-25 min, 90–100% B; and 25–30 min, 100% B. The injection volume was 5 *μ*L. The detection wavelengths of DAD were set at 210 nm, 225 nm, and 254 nm.

### 2.3. UPLC-DAD-QTOF-MS Analysis

The Waters ACQUITY UPLC instrument (Waters, MA, USA) coupled with Waters Synapt HDMS G1 (Waters, Manchester, UK) via an electrospray ionization (ESI) interface. The UPLC analytical conditions were the same as the UPLC analysis described above. The full scan mass spectra data were acquired in positive and negative ion modes. Acquisition parameters are as follows: capillary voltage was 3000 V for ESI (+) and 2600 V for ESI (−); cone voltage was 45 V; the ESI source temperature was 100°C; the desolvation temperature was 350°C; the nitrogen (N_2_) was used as desolvation gas at flow rates of 600 L/h for both ESI (+) and ESI (−); and the range of full scan was set at* m/z* 150–1000 Da. The version of analysis software was Mass Lynx V4.1.

### 2.4. Sample and Standard Solutions Preparation

The powder of LQC (0.4 g) was accurately weighed and extracted with 60% methanol-water (v/v) solution (20 mL) in an ultrasonic water bath for 30 min at room temperature. The supernatant solution was diluted with the same amount of water and then centrifuged for 10 min at 14,000 r/min. All the obtained solutions were filtered through 0.22 *μ*m syringe filter before the UPLC analysis.

Twelve standards were accurately weighed and dissolved in methanol to obtain stock solutions, respectively. A mixed stock solution of standards was prepared by adding a suitable volume of each stock solution to a 5 mL flask and diluted with 30% methanol-water solution at the concentration of 67.8 *μ*g/mL for salidroside, 109.65 *μ*g/mL for chlorogenic acid, 77.64 *μ*g/mL for forsythoside E, 106.47 *μ*g/mL for cryptochlorogenic acid, 62.57 *μ*g/mL for amygdalin, 31.96 *μ*g/mL for sweroside, 3.21 *μ*g/mL for hyperin, 8.5 *μ*g/mL for rutin, 67.34 *μ*g/mL for forsythoside A, 45.71 *μ*g/mL for phillyrin, 55.49 *μ*g/mL for rhein, and 84.35 *μ*g/mL for glycyrrhizic acid, respectively. The mixed stock solution was then serially diluted with 30% methanol-water solution to obtain five appropriate concentrations used for plotting standard curves. The lowest concentration of the mixture stock solution was further diluted to give a series of different concentrations for investigating the limits of detection (LODs) and limits of quantification (LOQs) of the 12 chemical constituents. All solutions were stored at 4°C until analysis.

### 2.5. Validation of the Quantitative Analysis

The UPLC-DAD method was evaluated with linearity, LOD, LOQ, precision, stability, repeatability, and recovery tests. The calibration curves were constructed with five different concentrations of chemical markers in triplicate. The LODs and LOQs were measured under the UPLC analytical conditions at a signal-to-noise (S/N) ratio of 3 and 10, respectively. For intraday and interday precisions test, the samples were analyzed by six repetitive injections within one day and once a day for three successive days, respectively. At room temperature, the stability of sample solution was evaluated by replicate injection at 0, 1, 2, 4, 6, 8, 10, 14, 24, and 48 h. In order to check the repeatability, six samples from the same source were investigated. Accurate amounts of the reference standards were added to 0.20 g powder of sample in sextuplicate. The resultant sample solutions were then extracted and quantified with the described method. The relative standard deviation (RSD) was used to evaluate the results.

## 3. Results and Discussion

### 3.1. Optimization of the Extraction and Chromatographic Conditions

A single-factor method was used to investigate the extraction effect of the extraction solvent (30%, 60%, and 90% methanol-water solution), extraction solvent ratio (1 : 50, 1 : 100, and 1 : 200 (w/v)), and extraction time (15 min, 30 min, and 45 min), respectively. By analyzing the extraction efficiency, 60% methanol-water solution, extraction solvent ratio at 1 : 100, and 30 min of ultrasonic time were selected as the eventual extraction conditions. The results are described in Table 1.

**Figure 1:**
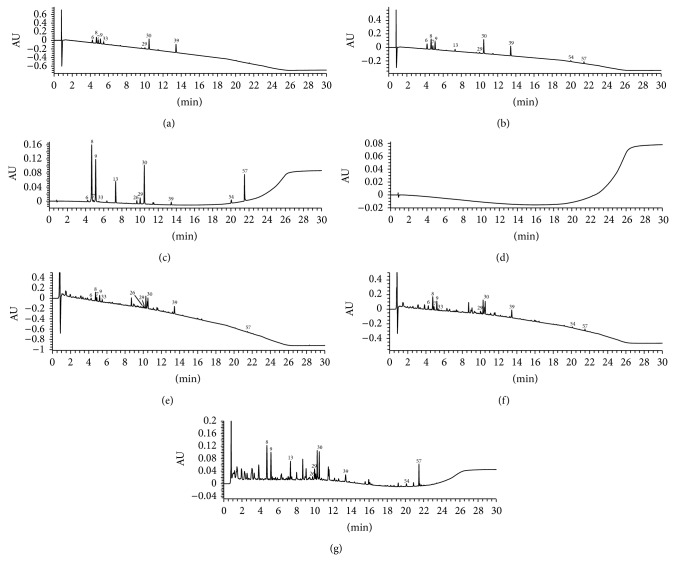
UPLC-DAD chromatograms of standard solution of 210 nm (a), 225 nm (b), 254 nm (c), negative sample solution (d), and sample solution of 210 nm (e), 225 nm (f), and 254 nm (g).

Due to the existence of acidic constituents in sample solutions, formic acid was added into the mobile phase which could inhibit the ionization of these acidic ingredients to improve the peak shape. The mobile phase systems (methanol-formic acid aqueous solution and acetonitrile-formic acid aqueous solution) and column temperature (40°C and 50°C) were investigated, which showed that methanol-0.1% formic acid aqueous solution as mobile phase with column temperature at 50°C could obtain the best chromatographic peak shape. Because the maximum absorptions of 12 reference compounds were different, three detection wavelengths were finally selected in order to achieve the goal of high detection sensitivity and little interference. Forsythoside E (peak 7), cryptochlorogenic acid (peak 9), amygdalin (peak 33), and phillyrin (peak 39) had satisfactory sensitivity at 210 nm, salidroside (peak 6), chlorogenic acid (peak 8), and rhein (peak 54) at 225 nm, and sweroside (peak 13), hyperin (peak 26), rutin (peak 29), forsythoside A (peak 30), and glycyrrhizic acid (peak 57) at 254 nm. The chromatograms are presented in Figure 1.

### 3.2. UPLC-DAD-QTOF-MS Analysis of Reference Compounds and LQC Samples

**Figure 2:**
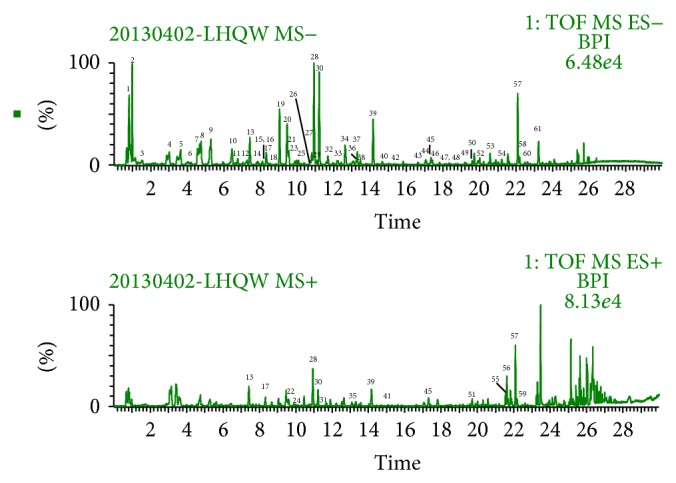
UPLC-QTOF-MS chromatograms of sample solution from negative ion mode and positive ion mode.

**Figure 3:**
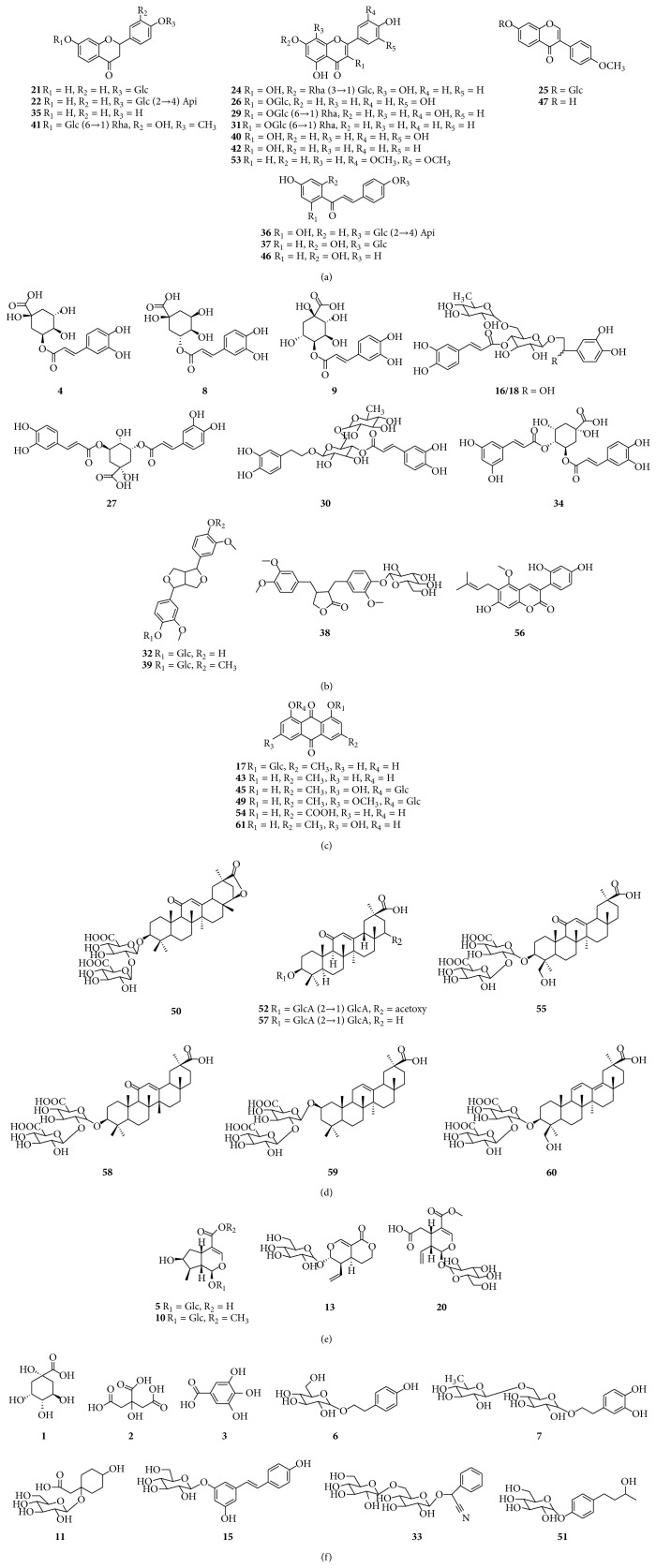
Chemical structures of the compounds identified from LQC (except for the 7 isomers).

As shown in Table 2, a total of 61 compounds were unambiguously or tentatively identified by comparing the retention times and accurate mass measurement with references or literature data. These compounds were divided into six types according to their structural characteristics including flavonoids, phenylpropanoids, anthraquinones, triterpenoids, iridoids, and other types. The structures of identified compounds are listed in Figure 3. Among them, twenty-seven compounds were further confirmed by comparing with standards. The total ion chromatograms are shown in Figure 2.

#### 3.2.1. Flavonoids

Seventeen flavonoids (Figure 3(a)) in LQC including flavone aglycones and glycosides were identified. They were mainly obtained from Lianqiao, Jinyinhua, Gancao, Hongjingtian, and Mahuang. Amongst them, liquiritin apioside (**22**), ononin (**25**), hyperin (**26**), rutin (**29**), liquiritigenin (**35**), isoliquiritin apioside (**36**), isoliquiritin (**37**), and formononetin (**47**) were unambiguously identified via the standards.

**Figure 4:**
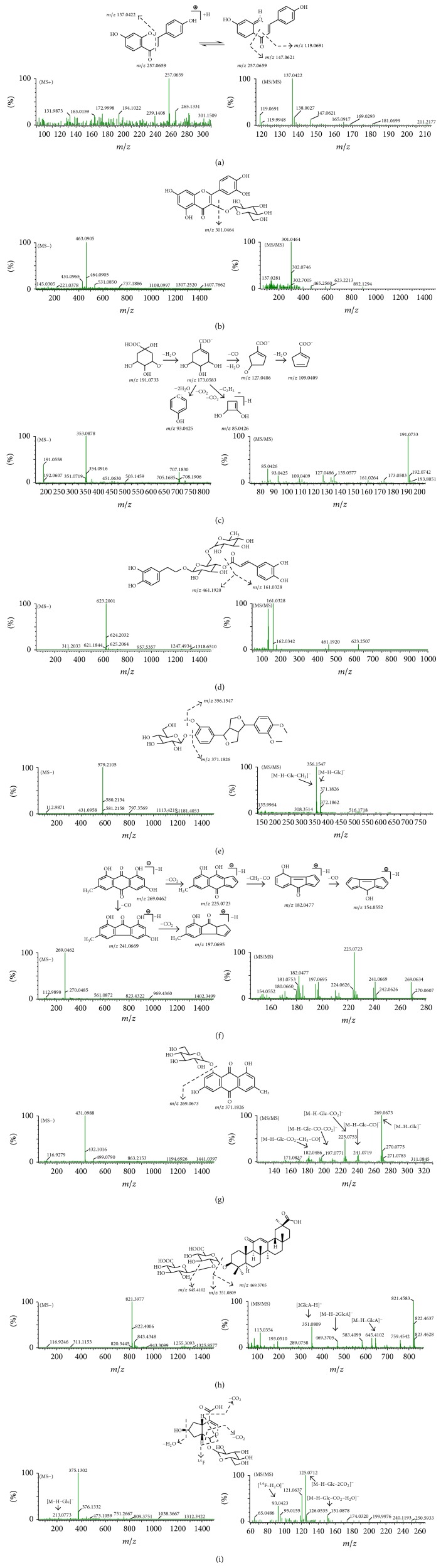
The MS spectra and fragmentation pathway of compound** 35** (a), compound** 26** (b), compound** 8** (c), compound** 30** (d), compound** 39** (e), compound** 61** (f), compound** 45** (g), compound** 57** (h), and compound** 5** (i).

In negative and positive ion modes, flavone aglycones mainly gain fragment ions by the reverse Diels-Alder (RDA) reaction and the loss of CO (28 Da). The characteristic fragmentation behavior of compound** 35** is shown in Figure 4(a) with high abundant fragmentation [M+H–VP (4-vinylphenol)]^+^ at* m/z* 137.0422. The abundance of fragment ions [M+H–RL (resorcinol)]^+^ at* m/z* 147.0621 and [M+H–RL–CO]^+^ at* m/z* 119.0691 is relatively lower. Compounds** 24**,** 40**,** 42,** and** 46 **were tentatively identified via comparing their exact molecular masses, MS/MS spectra data, and retention behaviors with literature data [13, 26, 39]. Flavone glycosides have the similar fragmentation pathways of simultaneous or successive loss of glucose (162 Da), rhamnose (146 Da), or apiose (132 Da). The fragmentation pathway of compound** 26** was exemplified in Figure 4(b) in negative ion mode. Compounds** 21**,** 23**,** 31**,** 41,** and** 53 **were tentatively identified by comparing the molecular mass and MS/MS data with literature data [25, 45].

#### 3.2.2. Phenylpropanoids

Fourteen phenylpropanoids (Figure 3(b)) including phenylpropionic acids, lignans, and coumarins were identified in LQC. They were mainly obtained from Lianqiao, Jinyinhua, and Gancao. Neochlorogenic acid (**4**), chlorogenic acid (**8**), cryptochlorogenic acid (**9**), 3,5-dicaffeoylquinic acid (**27**), forsythoside A (**30**), 3,4-dicaffeoylquinic acid (**34**), and phillyrin (**39**) were unambiguously characterized by comparing with standards.

In negative ion mode, phenylpropionic acids have the similar fragmentation pathways of simultaneous or successive loss of H_2_O (18 Da), CO (28 Da), and CO_2_ (44 Da). The fragmentation pathway of compound** 8 **[18], as the representative of phenylpropionic acids, is shown in Figure 4(c). Compound** 30** produced [M–H–Ca (caffeoyl)]^−^ at* m/z* 461.1920 and [Caffeic–H–H_2_O]^−^ at* m/z* 161.0328, as displayed in Figure 4(d). Lignans primarily generated [M+HCOO]^−^ in negative ion mode and further elimination of glucose (162 Da) produced aglycone. As shown in Figure 4(e), compound** 39 **produced characteristic fragments at* m/z* 371.1826 and* m/z* 356.1547 corresponding to [M–H–Glc (glucose)]^−^ and [M–H–Glc–CH_3_]^−^, respectively. Compounds** 16**,** 18**,** 19**,** 28**,** 32**,** 38, **and** 56 **were tentatively identified on the basis of the exact molecular formulae matching, fragmentation information, and retention behaviors as well as literature data [23, 24, 33, 36, 47].

#### 3.2.3. Anthraquinones

Eight anthraquinones (Figure 3(c)) in LQC were definitely or tentatively identified. All of them were derived from Dahuang. Chrysophanol glucoside (**17**), emodin-8-O-glucoside (**45**), rhein (**54**), and emodin (**61**) were confirmed via comparing with standard substances.

The characteristic fragmentation behavior of anthraquinones was the loss of CO_2_ (44 Da), CH_3_ (15 Da), and CO (28 Da) in negative ion mode. Typical compound** 61 **was used to explain the fragmentation pathway of anthraquinones presented in Figure 4(f). Compound** 45**, as shown in Figure 4(g), produced characteristic fragments at* m/z* 269.0673,* m/z* 241.0719,* m/z* 225.0753,* m/z* 197.0771, and* m/z* 182.0486 which corresponded to [M–H–Glc]^−^, [M–H–Glc–CO]^−^, [M–H–Glc–CO_2_]^−^, [M–H–CO–CO_2_]^−^, and [M–H–CO_2_–CH_3_–CO]^−^, respectively. Compounds** 12**,** 43**,** 44,** and** 49** were tentatively identified by comparing their accurate molecular masses and MS/MS fragment data with literature data [21, 40, 41].

#### 3.2.4. Triterpenoids

Eight triterpenoids (Figure 3(d)) in LQC were unambiguously or tentatively identified. All of them were derived from Gancao. Glycyrrhizic acid (**57**) was confirmed by comparing with standards. In negative ion mode, representative compound** 57 **yielded [M–H–H_2_O–CO_2_]^−^ at* m/z* 759.4524, [M–H–GlcA (glucuronic acid)]^−^ at* m/z* 645.4102, [M–H–2GlcA]^−^ at* m/z* 469.3705, [2GlcA–H]^−^ at* m/z* 351.0809, and [2GlcA–H–H_2_O–CO_2_]^−^ at* m/z* 289.0758, as shown in Figure 4(h). Compounds** 48**,** 50**,** 52**,** 55**,** 58**,** 59, **and** 60 **were tentatively identified by comparing their exact molecular masses and MS/MS spectral data with the literature data [51].

#### 3.2.5. Iridoids

Four iridoids (Figure 3(e)) in LQC including iridoid glycosides and secoiridoid glycosides were identified. All of them were derived from Jinyinhua. Loganic acid (**5**), sweroside (**13**), and secoxyloganin (**20**) were unambiguously identified via the standards.

The fragment ^1,4^F (fragment generated from the fracture of 1/4 bonds of iridoids) in the negative ion mode was identified as the characteristic fragment ion of iridoid glycosides. Meanwhile, iridoid glycosides would lose the functional groups such as H_2_O (18 Da), CO_2_ (44 Da), and glucose (162 Da). Typical compound** 5** gave [M–H–Glc–CO_2_–H_2_O]^−^ at* m/z* 151.0878, [M–H–Glc–2CO_2_]^−^ at* m/z* 125.0712, and [^1,4^F–H_2_O]^−^ at* m/z* 93.0423 [52], as presented in Figure 4(i). Compound** 10** was tentatively identified by comparing its exact molecular mass and MS/MS spectral data with the literature data [19].

#### 3.2.6. Other Types of Compounds

Compounds** 1**,** 2**,** 11**,** 14**,** 15,** and** 51 **were tentatively identified by comparing their exact molecular masses and MS/MS spectral data with the literature data except gallic acid (**3**), salidroside (**6**), forsythoside E (**7**), and amygdalin (**33**) which were identified via the standards (Figure 3(f)) [12, 53].

### 3.3. Methodological Validation of the Quantitative Analysis

As shown in Table 3, twelve standards were of good linearity with high correlation coefficient values over 0.9993. The LODs and LOQs were 0.051–1.71 *μ*g/mL and 0.16–5.69 *μ*g/mL, independently. Twelve analytes in sample solution were stable at room temperature within 48 h with the RSD less than 2.76%. The RSD values of intraday and interday precisions were less than 1.10% and 2.92%, respectively. The RSD of repeatability was less than 2.39%. The average recovery rates of 12 compounds ranged from 92.99% to 103.95% with the RSD less than 3.54%. All the results showed that the assay was satisfactory with high accuracy, good reproducibility, and high sensitivity which were beneficial to the analytical investigation and quality control for LQC.

### 3.4. Sample Analysis

Twelve representative compounds in 10 batches of LQC were quantified through the developed UPLC-DAD analytical method described above. The results are summarized in Table 4, which showed that the total concentrations of 12 quantitative compounds in different batches of the LQC varied narrowly; moreover, the 12 components differed greatly in their contents, which may be affected by the source of medicinal materials, the quality of the plant material, or the preparation technology. Among them, forsythoside A showed the highest amount (3164.55–2089.22 *μ*g/g) followed by amygdalin (2594.75–1623.14 *μ*g/g) and hyperin had the lowest amount at 100.80–62.56 *μ*g/g.

## 4. Conclusion

LQC is a commonly used Chinese medical preparation to treat viral influenza. To date, there has not been a systematical and comprehensive study on the chemical profiling and quality control method for LQC. Therefore, an accurate, sensitive, and reliable quality control procedure for LQC is in urgent need to be established. In our study, the chemical profile of LQC was thoroughly and systematically investigated by UPLC-DAD-QTOF-MS for the first time. Sixty-one compounds were unambiguously or tentatively identified. Based on the qualitative analysis, a UPLC-DAD method was established for quantitative analysis of 12 representative compounds in LQC, which has been demonstrated to be effective for the analysis of 10 batches of LQC. This developed method could be applied as an effective quality control procedure for LQC. In addition, this study would be a powerful reference for the identification of similar compounds presented here, such as flavonoids, phenylpropanoids, anthraquinones, triterpenoids, and iridoids by MS spectra.

## Figures and Tables

**Table 1:** Quantitative results of 12 compounds in LQC extracted by different methods.

Content (*μ*g/g)	Methods								
	30%^a^	60%	90%	60%	60%	60%	60%	60%	60%
	30 min^b^	30 min	30 min	30 min	30 min	30 min	15 min	30 min	45 min
	1 : 100^c^	1 : 100	1 : 100	1 : 50	1 : 100	1 : 200	1 : 100	1 : 100	1 : 100
Salidroside	1726.28	1701.25	1622.02	1522.31	1701.25	1656.32	1711.54	1701.25	1688.54
Chlorogenic acid	2444.97	2492.15	2216.89	2285.43	2492.15	2289.27	2492.62	2492.15	2552.17
Forsythoside E	1583.93	1620.78	451.91	1462.09	1620.78	1402.53	1627.01	1620.78	1579.63
Cryptochlorogenic acid	1862.98	1851.64	667.50	1703.05	1851.64	1771.79	1837.89	1851.64	1857.95
Amygdalin	1424.11	1455.39	1442.56	1268.93	1455.39	1298.11	1395.97	1455.39	1431.38
Sweroside	816.19	813.18	789.32	747.06	813.18	772.67	812.51	813.18	808.24
Hyperin	135.10	151.73	167.26	140.84	151.73	140.80	152.72	151.73	157.67
Rutin	122.00	121.17	115.22	106.11	121.17	116.67	117.09	121.17	116.62
Forsythoside A	2484.60	2536.34	2661.79	2285.76	2536.34	2396.35	2543.87	2536.34	2521.10
Phillyrin	1660.26	1521.45	1551.59	1410.19	1521.45	1390.12	1523.01	1521.45	1551.52
Rhein	803.13	1102.06	1370.11	937.63	1102.06	932.05	956.38	1102.06	1054.77
Glycyrrhizic acid	1530.49	1680.43	1594.37	1437.40	1680.43	1619.99	1674.81	1680.43	1665.42

^a^Extracting solvent: 30%, 60%, and 90% methanol-water solution.

^b^Ultrasonic time: 15 min, 30 min, and 45 min.

^c^Extraction solvent multiples: 1 : 50, 1 : 100, and 1 : 200 expressed 50, 100, and 200 times per gram of sample.

**Table 2:** Characterization of compounds in LQC by UPLC-DAD-QTOF-MS.

Peak number	*T*_*R*_ (min)	*λ*_max⁡_ (nm)	Formula	ES^−^ (*m*/*z*)		ES^+^ (*m*/*z*)			Identification	Reference
				[M–H]^−^ (ppm)	MS^2b^	[M + H]^+^(ppm)	[M + Na]^+^	MS^2b^		
**1**	0.817		C_7_H_12_O_6_	191.0555 (−0.5)					Quinic acid	[12]

**2**	0.982	223, 287	C_6_H_8_O_7_	191.0198 (3.1)	173.0099 [M–H–H_2_O]^−^, 111.0088 [M–H–2H_2_O–CO_2_]^−^				Citric acid	[13]

**3** ^ a^	1.545	216, 271	C_7_H_6_O_5_	169.0142 (3.0)	125.0241 [M–H–CO_2_]^−^				Gallic acid	[14]

**4** ^ a^	3.010	325	C_16_H_17_O_9_	353.0878 (1.4) 375.0695 [M–2H + Na]^−^, 707.1830 [2M–H]^−^	191.0497 [M–H–Ca]^−^, 179.0312 [Caffeic acid–H]^−^, 135.0359 [Caffeic acid–H–CO_2_]^−^				Neochlorogenic acid	[13]

**5** ^ a^	3.639	254	C_16_H_24_O_10_	375.1302 (2.9), 751.2667 [2M–H]^−^	213.0773 [M–H–Glc]^−^, 151.0878 [M–H–Glc–CO_2_–H_2_O]^−^, 125.0721 [M–H–Glc–2CO_2_]^−^				Loganic acid	[15]

**6** ^ a^	4.048	224, 278	C_14_H_20_O_7_	299.1133 (0.7)	179.0447 [M–H–C_8_H_8_O]^−^, 119.0246 [M–H–Glc–H_2_O]^−^				Salidroside	[16]

**7** ^ a^	4.557		C_20_H_30_O_12_	461.1674 (3.3)	315.1348 [M–H–Rha]^−^, 297.1589 [M–H–Rha–H_2_O]^−^, 153.0644 [M–H–Rha–Glc]^−^, 135.0573 [M–H–Rha–Glc–H_2_O]^−^	463.1220 (−4.3)	485.1568	137.0599 [M + H–Rha–Glc–H_2_O]^+^	Forsythoside E	[17]

**8** ^ a^	4.749	327	C_16_H_17_O_9_	353.0880 (2.0)	191.0556 [M–H–Ca]^−^, 179.0224 [Caffeic acid–H]^−^	355.1028 (−0.3)	377.0857		Chlorogenic acid	[18]

**9** ^ a^	5.281	325	C_16_H_17_O_9_	353.0880 (2.0)	179.0348 [Caffeic acid–H]^−^, 173.0461 [Quinic acid–H–H_2_O]^−^, 191.0417 [M–H–Ca]^−^				Cryptochlorogenic acid	[18]

**10**	6.435	270	C_17_H_26_O_10_	389.1096 (3.1)		391.2176 (−0.8)	413.1067	395.2354 [M + Na–H_2_O]^+^ 229.0835 [M + H–Glc]^+^	Loganin	[19]

**11**	6.749		C_14_H_23_O_9_	335.0776 (2.7)	173.0459 [M–H–Glc]^−^, 161.0373 [Glc–H–H_2_O]^−^, 133.0408 [Glc–H–H_2_O–CO]^−^	337.0883 (5.3)	359.0686	163.0419 [Glc + H–H_2_O]^+^	Rengynic acid-1′-O-β-D- glucoside	[20]

**12**	7.222		C_21_H_20_O_9_	415.1261 (5.1)			439.1207	255.0879 [M + H–Glc]^+^	Isomer of chrysophanol glucoside	[21]

**13** ^ a^	7.428	245	C_16_H_22_O_9_	357.1208 (6.2), 403.1263 [M + HCOO]^−^, 393.0938 [M + Cl]^−^		359.1337 (−1.4)	381.1154		Sweroside	[15]

**14**	7.859		C_14_H_23_O_9_	335.0871 (4.2)	193.0506, 161.0377, 133.0423	337.0774 (0.9)	359.0225	163.0416 [Glc + H–H_2_O]^+^	Isomer of rengynic acid-1′-O-β-D-glucoside	

**15**	8.106	306	C_20_H_22_O_8_	389.1458 (2.6), 435.1519 [M + HCOO]^−^	227.0943 [M–H–Glc]^−^				Polydatin	[22]

**16**	8.222		C_29_H_36_O_16_	639.2219 (−1.7)	477.1894 [M–H–Glc]^−^, 179.0546 [M–Rha–C_17_H_14_O_6_]^−^, 161.0328 [Glc–H–H_2_O]^−^,				R-suspensaside	[23]

**17** ^ a^	8.320		C_21_H_20_O_9_	415.1253 (3.1)	253.0542 [M–H–Glc]^−^	417.1394 (−0.7)	439.1200	255.0864 [M + H–Glc]^+^	Chrysophanol glucoside	[21]

**18**	8.874		C_29_H_36_O_16_	639.2002 (−2.7)	161.0328 [Glc–H–H_2_O]^−^, 179.0349 [M–Rha–C_17_H_14_O_6_]^−^				S-suspensaside	[24]

**19**	9.054		C_29_H_36_O_15_	623.1999 (−1.2)	311.2176	625.2137 (0.8)	647.1942	479.1562, 471.1479, 325.0925, 163.0394	Isomer of forsythoside A	

**20** ^ a^	9.470	235	C_17_H_24_O_11_	403.1261 (5.2)		405.1331 (−1.7)	427.1203	243.0871 [M + H–Glc]^+^	Secoxyloganin	[19]

**21**	9.552	276, 313	C_21_H_22_O_9_	417.1205 (4.6), 835.2331 [2M–H]^−^	255.0652 [M–H–Glc]^−^,		441.1132	257.0811 [M + H–Glc]^+^	Liquiritin	[25]

**22** ^ a^	9.886	277, 312	C_26_H_30_O_13_	549.1627 (3.5)		551.2823 (4.7)	573.1737	257.0822 [M + H–Api–Glc]^+^	Liquiritin apioside	[26]

**23**	9.995		C_26_H_30_O_13_	549.1629 (3.8)		551.3403 (−5.1)	573.1735	257.0815 [M + H–Api–Glc]^+^	Isomer of liquiritin apioside	[26]

**24**	10.088		C_27_H_30_O_16_	609.1844 (−1.8)			633.1712		Rhodiosin	[27]

**25** ^ a^	10.384	250, 300	C_22_H_22_O_9_	429.1410 (3.0)			453.1176	269.0974 [M + H–Glc]^+^	Ononin	[28, 29]

**26** ^ a^	10.722	360	C_21_H_20_O_12_	463.0905 (2.8)	301.0464 [M–H–Glc]^−^	465.1033 (5.2)	487.0333	303.0515 [M + H–Glc]^+^	Hyperin	[24]

**27** ^ a^	10.819	326	C_25_H_24_O_12_	515.1205 (2.9)	353.0751 [M–H–Ca]^−^	517.1296 (1.7)	539.1156	499.1187 [M + H–H_2_O]^+^, 163.0395 [M + H–Ca–Quinic acid]^+^	3,5-Dicaffeoylquinic acid	[30]

**28**	10.931		C_29_H_36_O_15_	623.1999 (−1.9)	461.1378, 161.0185		647.1941	471.1497, 325.0925, 163.0415	Isomer of forsythoside A	

**29** ^ a^	11.10	256, 354	C_27_H_30_O_16_	609.1465 (1.5)		611.1573 (3.3)	633.1404	465.0982 [M + H–Rha]^+^ 303.0495 [M + H–Rut]^+^	Rutin	[31]

**30** ^ a^	11.220	280	C_29_H_36_O_15_	623.2001 (−1.6)	461.1920 [M–H–Ca]^−^, 161.0328 [Caffeic acid–H–H_2_O]^−^		647.1927	163.0386 [Caffeic acid + H–H_2_O]^+^	Forsythoside A	[32]

**31**	11.589	283	C_27_H_30_O_15_	593.1534 (−1.3)	285.0677 [M–H–Rut]^−^	595.1694 (−0.7)		287.0936 [M + H–Rut]^+^	Kaempferol-3-O-rutinoside	[13]

**32**	11.692		C_19_H_35_O_16_	519.1884 (3.5), 565.1953 [M + HCOO]^−^	357.1366 [M–H–Glc]^−^		543.1855	359.1454 [M + H–Glc]^+^, 341.1411 [M + H–Glc–H_2_O]^+^	(+)-Pinoresinol-β-D-glucoside	[33]

**33** ^ a^	12.213	210, 262	C_20_H_27_NO_11_	456.1523 (3.7)		458.1641 (−4.6)		296.1124 [M + H–Glc]^+^, 162.0862 [M + H–Glc–HCT]^+^	Amygdalin	[13]

**34** ^ a^	12.659	326	C_25_H_24_O_12_	515.1162 (−5.4)	353.0786 [M–H–Ca]^−^	517.1348 (0.4)	539.1193	499.1248 [M + H–H_2_O]^+^, 163.0387 [M + H–Ca–Quinic acid]^+^	3,4-Dicaffeoylquinic acid	[30]

**35** ^ a^	13.082	276	C_15_H_12_O_4_	255.0654 (−1.2)		257.0659 (−0.8)		147.0621 [M + H–RL]^+^, 137.0422 [M + H–VP]^+^, 119.0691 [M + H–RL–CO]^+^	Liquiritigenin	[34]

**36** ^ a^	13.251	243, 372	C_26_H_30_O_13_	549.1621 (2.4)		551.1725 (3.4)	573.1563	419.1360 [M + H–Api]^+^, 257.0814 [M + H–Api–Glc]^+^	Isoliquiritin apioside	[35]

**37** ^ a^	13.300	242, 362	C_21_H_22_O_9_	417.1198 (2.9)	255.0518 [M–H–Glc]^−^	419.1314 (−6.7)	441.1150	257.0823 [M + H–Glc]^+^, 229.1357 [M + H–Glc–CO]^+^	Isoliquiritin	[26]

**38**	13.479	280	C_27_H_34_O_11_	579.2103 [M + HCOO]^−^			557.1987	552.2463 [M + NH_4_]^+^, 355.1516 [M + H–Glc–H_2_O]^+^	Arctiin	[36]

**39** ^ a^	14.161	277	C_27_H_34_O_11_	579.2106 [M + HCOO]^−^	371.1826 [M–H–Glc]^−^, 356.1547 [M–H–Glc–H_2_O]^−^	552.2444 [M + NH_4_]^+^	557.1991	373.1563 [M + H–Glc]^+^, 355.1544 [M + H–Glc–H_2_O]^+^	Phillyrin	[37, 38]

**40**	14.678	372, 255	C_15_H_10_O_7_	301.0355 (2.3)	151.0137 [M–H–C_8_H_6_O_3_]^−^	303.0520 (4.9)			Quercetin	[39]

**41**	15.039	284	C_28_H_34_O_15_	609.1522 (1.3)	301.0582 [M–H–Rut]^−^				Hesperidin	[13]

**42**	15.402	265, 366	C_15_H_10_O_6_	285.0406 (2.5)		287.0721 (4.5)		153.0469 [M + H–HEP]^+^	Kaempferol	[13]

**43**	16.627	255	C_15_H_10_O_4_	253.0507 (2.4)	225.0741 [M–H–CO]^−^, 210.0494 [M–H–CO–CH_3_]^−^, 182.0542 [M–H–2CO–CH_3_]^−^, 154.0553 [M–H–3CO–CH_3_]^−^				Chrysophanol	[40]

**44**	17.035		C_15_H_10_O_4_	253.0512 (4.3)	225.0752, 210.0474, 182.0485, 154.0531				Isomer of chrysophanol	[40]

**45** ^ a^	17.347	254, 287, 426	C_21_H_20_O_10_	431.0989 (2.6)	269.0673 [M–H–Glc]^−^, 241.0719 [M–H–Glc–CO]^−^, 225.0753 [M–H–Glc–CO_2_]^−^, 210.0578 [M–H–Glc–CO_2_–CH_3_]^−^, 197.0771 [M–H–Glc–CO–CO_2_]^−^, 182.0486 [M–H–Glc–CO_2_–CH_3_–CO]^−^		455.1010	271.0715 [M + H–Glc]^+^	Emodin-8-O-glucoside	[41]

**46**	17.396	240, 330, 395	C_15_H_12_O_4_	255.0639 (−7.1)		257.0812 (−0.8)			Isoliquiritigenin	[26]

**47** ^ a^	18.385	250, 304	C_16_H_12_O_4_	267.0653 (−1.5)		269.0827 (4.8)	291.0751		Formononetin	[42]

**48**	18.804			895.4082 (2.8)	719.3849 [M–H–GlcA]^−^ 351.0630 [2GlcA–H]^−^	897.4107 (1.2)	919.3925		22β-Acetoxy licorice saponin B2/uralsaponin F	[43]

**49**	19.194	271, 421	C_22_H_22_O_10_	445.1168 (−5.6)	283.0632 [M–H–Glc]^−^		469.1128	285.0775 [M + H–Glc]^+^	Physcion-8-O-β-D-glucopyranoside	[41]

**50**	19.603	248	C_42_H_60_O_16_	819.3843 (0.6)	351.0699 [2GlcA–H]^−^	821.3994 (−0.1)	843.3996	645.3612 [M + H–GlcA]^+^, 469.3302 [M + H–2GlcA]^+^, 451.3211 [M + H–2GlcA–H_2_O]^+^	Licorice saponin E2	[35]

**51**	19.704		C_16_H_24_O_7_	327.2180 (2.8)	164.9664 [M–H–Glc]^−^		351.2133		Rhododendrol-4′-O-β-D-glucopyranoside	[44]

**52**	20.000	254	C_44_H_64_O_18_	879.4062 (1.3)	351.0806 [M–H–22AG]^−^	881.4170 (−0.1)		705.3897 [M + H–GlcA]^+^, 529.3588 [M + H–2GlcA]^+^, 511.3395 [M + H–2GlcA–H_2_O]^+^	22-Acetoxyglycyrrhizin	[42]

**53**	20.573	352	C_17_H_14_O_7_	329.2341 (3.9)			353.2280	301.1443 [M + H–CH_2_O]^+^, 287.2002 [M + H–CO_2_]^+^, 259.1248 [M + H–CO_2_–CO]^+^	Tricin	[45]

**54** ^ a^	21.199	260	C_15_H_8_O_6_	283.0262 (6.7)	239.0359 [M–H–CO_2_]^−^	285.0515 (−13.0)			Rhein	[46]

**55**	21.540	253		837.3977 (1.2)	819.4404 [M–H–H_2_O]^−^, 661.4100 [M–H–GlcA]^−^, 351.0815 [2GlcA–H]^−^, 289.0840 [2GlcA–H–H_2_O–CO_2_]^−^	839.4061 (−0.5)	861.3890	663.3868 [M + H–GlcA]^+^, 487.3395 [M + H–2GlcA]^+^, 469.3286 [M + H–2GlcA–H_2_O]^+^	Licorice saponin G2	[42]

**56**	21.626	236, 327, 370, 384	C_21_H_20_O_6_	367.1208 (7.1)	309.1566 [M–H–CO–CH_2_O]^−^, 265.0635 [M–H–CO–CH_2_O–CO_2_]^−^, 221.0221 [M–H–CO–CH_2_O–2CO_2_]^−^	369.1369 (−7.6)	391.1176		Glycycoumarin	[47]

**57**	22.087	249	C_42_H_62_O_16_	821.3977 (1.7)	803.4501 [M–H–H_2_O]^−^, 759.4542 [M–H–H_2_O–CO_2_]^−^, 645.4102 [M–H–GlcA]^−^, 627.4000 [M–H–GlcA–H_2_O]^−^, 469.3705 [M–H–2GlcA]^−^, 351.0809 [2GlcA–H]^−^, 289.0758 [2GlcA–H–H_2_O–CO_2_]^−^	823.4128 (1.5)	845.4557	647.3797 [M + H–GlcA]^+^, 471.3480 [M + H–2GlcA]^+^, 453.3365 [M + H–2GlcA–H_2_O]^+^	Glycyrrhizic acid	[48]

**58**	22.188	252	C_42_H_62_O_16_	821.3986 (−1.1)	351.0768 [M–H–GA]^−^	823.4134 (−2.1)	845.4127	647.3823 [M + H–GlcA]^+^, 471.3365 [M + H–2GlcA]^+^, 453.3324 [M + H–2GlcA–H_2_O]^+^	Licorice saponin H2	[26]

**59**	22.389	248	C_42_H_64_O_15_	807.4197 (−0.6)	745.4601 [M–H–Glc]^−^, 631.4210 [M–H–GlcA]^−^, 351.0699 [M–H–GA]^−^	809.4410 (−0.9)	831.4138		Licorice saponin B2	[49]

**60**	22.623	252	C_42_H_62_O_16_	821.3997 (0.2)	351.0837 [M–H–GA]^−^	823.4105 (−1.3)	845.3900	647.3838 [M + H–GlcA]^+^, 471.3288 [M + H–2GlcA]^+^, 453.3279 [M + H–2GlcA–H_2_O]^+^	Licorice saponin K2	[50]

**61** ^ a^	23.230	288, 439	C_15_H_10_O_5_	269.0462 (4.5)	241.0669 [M–H–CO]^−^, 225.0723 [M–H–CO_2_]^−^, 210.0427 [M–H–CO_2_–CH_3_]^−^, 197.0695 [M–H–CO–CO_2_]^−^, 182.0477 [M–H–CO_2_–CH_3_–CO]^−^, 154.0552 [M–H–CO_2_–CH_3_–2CO]^−^				Emodin	[40]

^a^Compared with reference compounds.

^b^22AG: 22-acetoxyglycyrrhizin; Api: apiose; Ca: caffeoyl; GA: glycyrrhetinic acid; Glc: glucose; GlcA: glucuronic acid; HCT: 4-(hydroxymethyl)cyclobutane-1,2,3-triol; HEP: 4-(hydroxyethynyl) phenol; Rha: rhamnose; RL: resorcinol; Rut: rutinose; VP: 4-vinylphenol.

**Table 3:** Linear regression, LODs and LOQs, intraday and interday precisions, repeatability, stability, and recovery for 12 compounds.

Compound	Regression equation^a^ (*n* = 3)	*R* ^2^	Linear range (*μ*g/mL)	LOD^b^ (μg/mL)	LOQ^c^ (μg/mL)	Intraday (RSD, %) (*n* = 6)	Interday (RSD, %) (*n* = 3)	Repeatability (*n* = 6)		Stability (RSD, %)	Recovery (*n* = 6)				
								Mean (*μ*g/g)	RSD (%)		Original (*μ*g)	Spiked (μg)	Detected (μg)	Recovery (%)	RSD (%)
Salidroside	*y* = 13980*x* − 11265	0.9999	5.27–84.35	1.06	3.50	0.12	0.26	1779.55 ± 10.92	0.61	1.38	355.91	354.20	707.50	99.28	1.24

Chlorogenic acid	*y* = 26510*x* + 3673.3	0.9998	6.85–109.56	1.71	5.69	0.77	2.12	2473.71 ± 12.74	0.51	2.54	494.74	493.85	959.90	94.19	2.42

Forsythoside E	*y* = 11793*x* + 5016.8	0.9995	4.85–77.64	1.21	4.04	0.67	2.25	1754.03 ± 18.72	1.07	2.67	351.81	351.05	677.26	92.99	3.54

Cryptochlorogenic acid	*y* = 21498*x* − 5018.9	0.9997	6.65–106.47	1.66	5.53	1.10	1.63	2029.49 ± 20.65	1.02	2.46	405.90	406.60	784.00	93.10	1.43

Amygdalin	*y* = 10530*x* − 3464.7	0.9998	3.91–62.57	0.98	3.26	0.79	0.93	1485.38 ± 25.85	1.74	2.48	297.08	298.62	576.39	93.58	3.32

Sweroside	*y* = 20593*x* − 6003.4	0.9999	2.00–31.96	0.25	0.80	0.11	0.33	798.06 ± 2.93	0.37	0.48	159.61	159.51	325.43	103.95	0.41

Hyperin	*y* = 27773*x* − 879.41	0.9999	0.20–3.21	0.051	0.16	0.78	0.62	92.03 ± 0.80	0.87	0.83	18.41	18.24	37.07	102.29	1.82

Rutin	*y* = 30768*x* − 3783.7	0.9993	0.53–8.50	0.072	0.21	0.41	2.24	184.15 ± 3.06	1.66	1.57	36.83	36.81	72.50	96.90	2.34

Forsythoside A	*y* = 8546.6*x* − 6024.5	0.9999	4.21–67.34	0.30	1.05	0.20	2.92	2484.99 ± 18.43	0.74	1.95	497.00	495.95	997.33	100.89	1.49

Phillyrin	*y* = 30390*x* + 5282.8	0.9999	2.86–45.71	0.71	2.36	0.79	1.63	1577.80 ± 10.11	0.64	2.76	315.56	316.72	623.99	100.25	1.60

Rhein	*y* = 7473.6*x* − 5172.7	0.9996	3.47–55.49	0.87	2.88	0.40	0.28	681.92 ± 16.32	2.39	0.97	136.38	136.27	273.48	100.61	1.61

Glycyrrhizic acid	*y* = 10296*x* − 6531.3	0.9999	5.27–84.35	0.092	0.33	0.34	2.09	1622.75 ± 17.59	1.08	2.15	324.55	323.939	652.16	101.31	3.27

^a^ *y* is the peak area; *x* is the concentration of standard solutions.

^b^LOD refers to the limits of detection, *S*/*N* = 3.

^c^LOQ refers to the limits of quantity, *S*/*N* = 10.

**Table 4:** Contents of the 12 compounds in 10 batches.

Sample	Compound (mean ± SD) (*μ*g/g)												Total	RSD (%)
	Salidroside	Chlorogenic acid	Forsythoside E	Cryptochlorogenic acid	Amygdalin	Sweroside	Hyperin	Rutin	Forsythoside A	Phillyrin	Rhein	Glycyrrhizic acid		
Lot.1	1740.57 ± 30.70	2159.11 ± 12.47	1888.99 ± 7.95	1464.88 ± 10.22	2109.85 ± 31.27	830.23 ± 8.44	67.06 ± 0.68	156.22 ± 1.11	3163.84 ± 33.14	1599.20 ± 12.29	570.70 ± 2.23	1028.45 ± 4.21	16779.10	10.03
Lot.2	1697.04 ± 12.54	1810.69 ± 11.73	1424.67 ± 38.04	1167.41 ± 8.35	1623.14 ± 25.14	650.95 ± 7.21	68.07 ± 1.37	106.85 ± 2.82	2497.34 ± 27.21	1220.59 ± 35.57	554.26 ± 8.27	837.16 ± 11.65	13658.17	
Lot.3	1456.36 ± 22.08	1941.91 ± 44.94	1477.93 ± 29.66	1232.30 ± 29.88	1641.25 ± 34.31	681.53 ± 6.35	72.66 ± 2.05	110.31 ± 2.16	2089.22 ± 36.09	1314.99 ± 31.21	617.91 ± 10.70	904.75 ± 15.73	13541.12	
Lot.4	1520.75 ± 8.91	1993.38 ± 50.86	1501.09 ± 42.60	1409.15 ± 6.32	2147.01 ± 60.37	743.98 ± 18.00	62.56 ± 1.28	100.86 ± 2.39	2652.53 ± 67.03	1139.47 ± 24.08	696.42 ± 18.76	985.60 ± 22.85	14925.80	
Lot.5	1593.12 ± 35.00	2081.89 ± 50.77	1626.62 ± 48.29	1523.11 ± 43.22	2282.76 ± 46.19	777.98 ± 4.18	75.89 ± 1.69	121.06 ± 2.41	3072.10 ± 44.89	1477.18 ± 25.48	686.35 ± 16.44	984.79 ± 29.13	16302.85	
Lot.6	1821.34 ± 48.55	2436.69 ± 31.00	2025.28 ± 43.31	1494.76 ± 12.65	2377.04 ± 63.64	860.29 ± 13.37	100.80 ± 1.87	145.77 ± 2.94	3680.72 ± 92.85	1991.09 ± 18.29	648.86 ± 3.91	1057.29 ± 30.41	18639.93	
Lot.7	1474.32 ± 36.85	2222.61 ± 38.27	1372.97 ± 36.85	1727.36 ± 16.40	2594.75 ± 50.67	854.30 ± 19.31	77.25 ± 2.14	156.90 ± 4.65	2987.54 ± 36.76	1168.12 ± 12.70	551.26 ± 5.18	1227.74 ± 20.24	16415.12	
Lot.8	1598.66 ± 19.51	2183.54 ± 15.11	1589.79 ± 19.71	1721.26 ± 11.36	2494.81 ± 19.61	824.71 ± 4.47	81.26 ± 0.69	174.62 ± 0.86	3164.55 ± 36.81	1277.14 ± 11.04	716.94 ± 5.74	1220.87 ± 9.89	17048.15	
Lot.9	1535.78 ± 30.36	1961.72 ± 22.33	1789.55 ± 28.12	1247.34 ± 36.59	1824.87 ± 35.66	696.40 ± 7.08	73.33 ± 0.46	136.23 ± 2.51	2823.09 ± 15.19	1495.85 ± 17.76	625.10 ± 11.44	891.45 ± 10.60	15100.71	
Lot.10	1836.41 ± 29.14	2216.36 ± 8.18	1500.75 ± 8.06	1670.72 ± 23.19	2281.74 ± 14.32	809.41 ± 3.13	75.33 ± 0.85	134.19 ± 1.24	3010.34 ± 12.64	1204.92 ± 28.26	891.01 ± 9.99	1176.72 ± 6.68	16807.90	
